# A Simplified CT Score for Thrombus Burden in Acute Pulmonary Embolism: Clinical Correlation and Reproducibility

**DOI:** 10.3390/jimaging12070327

**Published:** 2026-07-19

**Authors:** Ignacio Díaz-Lorenzo, Rio Jorge Aguilar Torres, Paloma Caballero Sanchez-Robles, Raquel Caminero Garcia, Alfonso Canabal Berlanga, Alfonsa Friera Reyes, Alberto Alonso-Burgos

**Affiliations:** 1 Radiology Department, University Hospital La Princesa, Universidad Autónoma de Madrid, 28006 Madrid, Spain; pcaballeros@salud.madrid.org (P.C.S.-R.); alfonsa.friera@salud.madrid.org (A.F.R.); 2 Cardiology Department, University Hospital La Princesa, 28006 Madrid, Spain; riojorge.aguilar@salud.madrid.es; 3 Emergency Department, University Hospital La Princesa, 28006 Madrid, Spain; raquel.caminero@salud.madrid.org; 4 Critical Care Department, University Hospital La Princesa, 28006 Madrid, Spain; alfonso.canabal@salud.madrid.org; 5 Radiology Department, Clínica Universidad de Navarra, 28027 Madrid, Spain; alonso@unav.es

**Keywords:** pulmonary embolism, computed tomography pulmonary angiography, thrombus burden, risk stratification, right ventricular dysfunction

## Abstract

(1) Objectives: In acute pulmonary embolism (PE), detailed thrombus burden scores are often complex and time-consuming, limiting their integration into urgent radiology reports. We evaluated a simplified modified Ghanima score (GmScore and GmS) designed to provide a structured estimate of thrombus burden and assessed its clinical correlation and reproducibility. (2) Methods: In this retrospective single-center study, 132 consecutive patients with confirmed acute PE were classified according to the modified GmScore: GmS1 (segmental), GmS2 (lobar), and GmS3 (main pulmonary arteries), considering luminal obstruction ≥ 50%. European Society of Cardiology (ESC) risk category, simplified Pulmonary Embolism Severity Index (sPESI), CT right-to-left ventricular (RV/LV) ratio, echocardiographic right ventricular dysfunction, and 30-day mortality were recorded. Inter- and intraobserver agreement were assessed using weighted kappa. (3) Results: In 132 patients (mean age 64.8 ± 16.5 years; 77 men), a significant clinical gradient was observed across GmScore categories. ESC intermediate–high/high risk occurred in 0% of GmS1 and 95.6% of GmS2–3 patients (*p* < 0.001). The median RV/LV ratio increased progressively (0.76, 1.58, and 1.79 for GmS1–3; *p* < 0.001), with a strong correlation between the GmScore and RV/LV (Spearman ρ = 0.75). GmS2 and GmS3 showed no significant difference in ventricular repercussion (*p* = 0.938), whereas GmS1 differed markedly. Using GmS ≥ 2 to identify ESC intermediate–high/high risk yielded 100% sensitivity and negative predictive value. Interobserver agreement was excellent (κ = 0.92). Thirty-day mortality was 0% in GmS1, 2.0% in GmS2, and 14.6% in GmS3 (*p* = 0.005). (4) Conclusions: The modified GmScore is a simple, reproducible CT-based descriptor that aligns closely with right ventricular repercussion and ESC risk stratification.

## 1. Introduction

The way radiologists convey information to referring physicians remains a challenge in everyday clinical practice. The traditional radiology report, usually written in a narrative format, allows flexibility and nuance but introduces considerable variability that may limit its clinical usefulness, particularly when rapid decisions are required [1]. Report standardization has been proposed to improve communication, and several studies have shown that clinicians prefer reports that are clear, concise, and structured, especially in urgent settings [2]. However, implementing structured approaches can be difficult in high-pressure clinical environments where time is limited.

This issue is particularly relevant in pulmonary embolism (PE). In this condition, the radiology report not only confirms the diagnosis but may also influence critical therapeutic decisions. Current PE management relies on risk stratification that integrates clinical, laboratory, and imaging data to identify patients at risk of deterioration or early death [3]. CT pulmonary angiography is the diagnostic reference standard and provides important information on cardiac involvement, including the right-to-left ventricular (RV/LV) ratio, which has been consistently associated with worse outcomes and higher mortality [4,5].

Nevertheless, information on thrombus burden and distribution is not always translated into a practical and clinically usable format within the radiology report. Although several indices have been proposed to quantify pulmonary arterial obstruction, their complexity and the need for systematic segmental analysis limit their use in emergency settings, where reports must summarize findings clearly and reproducibly to support clinical decision-making. In this context, Ghanima proposed a simple approach based on the most proximal location of the thrombus, hypothesizing that involvement of larger vessels might reflect greater severity [6]. However, anatomical location alone does not consistently correlate with clinical outcomes, and its usefulness depends on how the information is incorporated into radiological communication [7].

Against this background, a key gap remains: existing clot burden scores are rarely integrated into emergency radiology reports because their complexity makes them impractical under time pressure. A simplified, location-based descriptor that requires no segmental analysis and aligns with clinical risk stratification could address this unmet need. We hypothesized that a modified version of the Ghanima score (GmScore), incorporating a threshold of ≥50% luminal obstruction, would correlate with established clinical risk categories and right ventricular strain parameters while maintaining excellent interobserver reproducibility.

The aim of this study was to evaluate the clinical correlation and interobserver reproducibility of a simplified CT-based score for thrombus burden in acute pulmonary embolism.

## 2. Materials and Methods

This retrospective single-center study was approved by the institutional review board, and the requirement for informed consent was waived. This study was conducted in accordance with the Declaration of Helsinki principles.

### 2.1. Modified Ghanima Score

The original score described by Ghanima classifies acute pulmonary embolism according to the most proximal thrombus location on CT pulmonary angiography into four anatomical levels: subsegmental arteries (grade 1), segmental arteries (grade 2), lobar arteries (grade 3), and main pulmonary arteries (grade 4), assigning greater severity to larger-caliber vessels [6]. Although simple and reproducible [8], it does not account for the degree of luminal obstruction or its hemodynamic impact.

**Figure 1:**
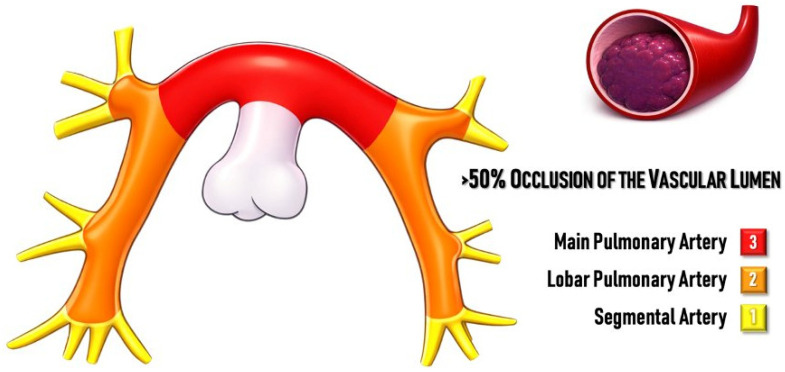
Diagram of the GmScore for the simplified quantification of thrombus burden in acute pulmonary embolism. The score is based on the most proximal anatomical location of a thrombus causing ≥50% luminal occlusion: main pulmonary artery (GmS3), lobar arteries (GmS2), and segmental arteries (GmS1). The color coding (red, orange, and yellow, respectively) reflects the anatomical hierarchy and the estimated thrombus burden.

At our institution, a modified version was adopted based on two criteria. First, only filling defects with luminal occupation ≥ 50% were considered and visually assessed as a filling defect occupying more than half of the vessel cross-sectional area on axial images; when visual assessment was uncertain, a caliper measurement of the filling defect and the vessel lumen was performed on the axial plane to confirm the threshold, following the approach proposed by Mastora, who demonstrated that this threshold is associated with increased mean pulmonary arterial pressure (45 ± 15 mmHg vs. 31 ± 11 mmHg in obstructions < 50%; *p* < 0.01), highlighting its pathophysiological relevance for right ventricular afterload [3,9]. Second, the largest involved vessel was prioritized regardless of the number of affected branches. The modified Ghanima score (GmScore, GmS) includes three categories: GmS3 (main pulmonary arteries), GmS2 (lobar arteries), and GmS1 (segmental arteries), provided that luminal occupation exceeds 50% (Figure 1). Subsegmental arteries were not considered, and the number of involved vessels was not taken into account when a larger vessel was affected.

**Figure 2:**
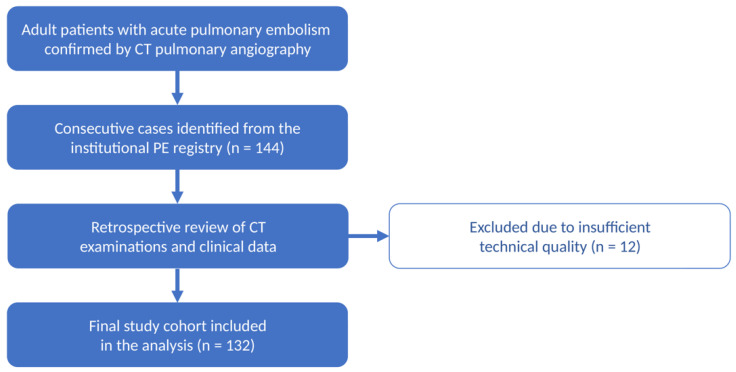
Flowchart of patient selection. Adult patients with acute pulmonary embolism confirmed by CT pulmonary angiography were consecutively identified from the institutional pulmonary embolism registry. CT examinations and clinical data were retrospectively reviewed. Recruitment for each anatomical category was closed once the predefined sample size of 30–40 patients per group had been reached, in accordance with the statistical requirements for association analysis and interobserver agreement estimation. All eligible cases were included in the final study cohort (*n* = 132). PE = Pulmonary embolism.

This single-center retrospective observational study was based on a systematically maintained patient registry. Study variables, including the definition of the GmScore, were established at study conception. Adult patients with a diagnosis of acute pulmonary embolism confirmed by CT pulmonary angiography were included between January 2021 and November 2025 (*n* = 144). CT pulmonary angiography acquisition and contrast administration protocols are detailed in Table 1. Patients were enrolled consecutively within each GmScore category until the predefined sample size was reached, at which point recruitment for that category was closed independently of the others. Given the lower prevalence of proximal PE, GmS3 was the last category to complete enrolment. Twelve studies were excluded due to insufficient technical quality for GmScore classification. The final cohort included 41 patients in GmS1, 50 in GmS2, and 41 in GmS3 (*n* = 132). The patient selection process is shown in Figure 2.

Clinical, imaging, and echocardiographic data were collected in a structured manner with the predefined objective of subsequent analysis. Comorbid conditions were recorded but not included as independent variables in the analysis, as this study was designed to evaluate the imaging descriptor rather than to develop a multivariable prognostic model. Clinical risk was captured through the sPESI, which incorporates the most relevant comorbidities for short-term prognosis in acute pulmonary embolism. All CT pulmonary angiograms were reviewed according to this protocol. Risk stratification according to the European Society of Cardiology (ESC), into four categories (low, intermediate–low, intermediate–high, and high), was determined during the initial clinical assessment, as was the simplified Pulmonary Embolism Severity Index (sPESI), by the treating physician [3]. Echocardiographic data (right ventricular dysfunction, TAPSE—tricuspid annular plane systolic excursion < 16 mm, and McConnell’s sign) were obtained in all patients: immediately in those classified as intermediate–high or high risk, and within the first 72 h in those at intermediate–low or low risk. This difference in timing represents a potential source of bias, as echocardiographic parameters obtained earlier may reflect a more acute haemodynamic state.

**Figure 3:**
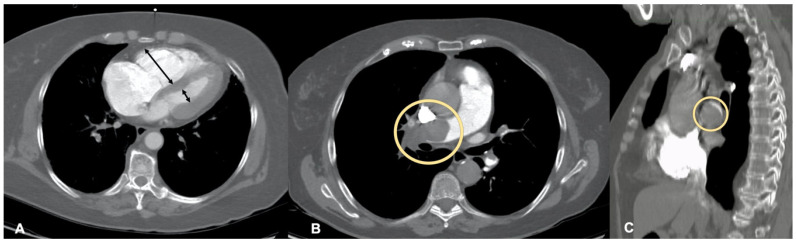
(**A**) Axial CT pulmonary angiography image showing right ventricular dilatation with an RV/LV ratio > 1 (4.56/1.49 cm = 3.06; black arrows), consistent with right ventricular strain. (**B**,**C**) Axial and sagittal images demonstrating a thrombus located in the right main pulmonary artery, occupying >50% of the vessel lumen (yellow circles). Findings correspond to GmS3. RV/LV = right ventricle to left ventricle diameter ratio.

A structured report was used, including: (1) description of filling defects and degree of luminal occupation (≥50% or <50%); (2) classification according to the GmScore; (3) axial RV/LV ratio; and (4) other relevant findings. The RV/LV ratio was measured as the maximum diameter of each ventricle on the axial slice showing the largest dimension, using a threshold of >1 to define right ventricular dilatation (Figure 3) [3]. Interobserver agreement was assessed in all studies through independent readings by an Emergency Radiologist and a Vascular Radiologist, both with more than 10 years of experience. Intraobserver agreement was evaluated by a second blinded reading of 30 randomly selected studies, with a minimum interval of four weeks between assessments.

### 2.2. Sample Size

Given that the study design was comparative and associative, focused on predefined anatomical categories rather than on the development of a multivariable predictive model, the events-per-variable criterion was not applicable. A sample of 30–40 patients per group was considered sufficient to obtain stable estimates of association [10] and a precise estimation of the kappa coefficient across three ordinal categories [11]. Recruitment for each category was closed once this predefined sample size had been reached. The final cohort included 41 patients in GmS1, 50 in GmS2, and 41 in GmS3 (*n* = 132).

### 2.3. Statistical Analysis

Continuous variables are presented as mean ± standard deviation or median (interquartile range), as appropriate; categorical variables are expressed as number and percentage. Comparisons across GmScore categories were performed using the Kruskal–Wallis test for continuous variables and Fisher’s exact test for categorical variables. For variables with a natural order, the Cochran–Armitage test for trend was applied. Post hoc pairwise comparisons of the RV/LV ratio were conducted using Dunn’s test with Bonferroni correction.

A dichotomous analysis comparing GmS1 versus GmS2 + GmS3 was performed, with calculations of sensitivity, specificity, and positive and negative predictive values, each with 95% confidence intervals, for the identification of intermediate–high or high ESC risk. The association between the GmScore and the continuous RV/LV ratio was assessed using Spearman’s correlation coefficient. Observer agreement was quantified using the quadratic weighted kappa coefficient with 95% confidence intervals. Proportions are reported with 95% confidence intervals calculated using the exact Clopper–Pearson method. A *p* < 0.05 was considered statistically significant. Statistical analyses were performed using Python (version 3.12.12; Python Software Foundation).

This study was conducted and reported in accordance with the STROBE (Strengthening the Reporting of Observational Studies in Epidemiology) guidelines. The STROBE checklist is provided as Supplementary Material.

## 3. Results

A total of 132 patients with confirmed pulmonary embolism were included: GmS1 (*n* = 41), GmS2 (*n* = 50), and GmS3 (*n* = 41). The continuous RV/LV ratio on CT was available in 116 patients (88%). The remaining 16 patients were referred from an external center, where images could be reviewed but RV/LV ratio measurements could not be performed (8 in GmS2 and 8 in GmS3). Baseline demographic characteristics were comparable across groups, with no significant differences in mean age (65.7 ± 18.7 years in GmS1; 64.9 ± 15.0 in GmS2; 63.8 ± 16.4 in GmS3; *p* = 0.806) or sex distribution (Table 2).

### 3.1. Inter- and Intraobserver Variability

Interobserver agreement, assessed through independent readings of all studies, was excellent (quadratic weighted kappa = 0.92; 95% CI: 0.87–0.97). Intraobserver agreement, evaluated in a random subsample of 30 studies with a minimum interval of four weeks between readings, was similarly excellent (quadratic weighted kappa = 0.92; 95% CI: 0.81–1.00). In both analyses, discrepancies occurred only between adjacent categories, with no cases of disagreement between GmS1 and GmS3.

### 3.2. Clinical Gradient

**Figure 4:**
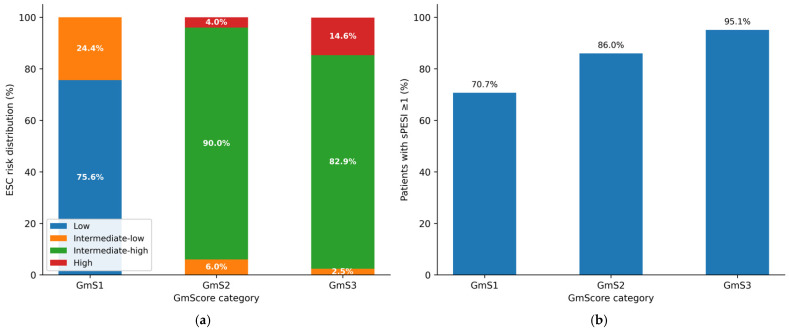
Association between GmScore and clinical risk classifications. (**a**) Distribution of European Society of Cardiology (ESC) risk categories within each GmScore group. (**b**) Proportion of patients with simplified Pulmonary Embolism Severity Index (sPESI) ≥ 1 according to GmScore category.

A progressive and statistically significant increase in clinical severity was observed across GmScore categories (Table 1, Figure 4). The proportion of patients with sPESI ≥ 1 rose from 70.7% in GmS1 to 86.0% in GmS2 and 95.1% in GmS3 (χ^2^ for trend = 9.34; *p* = 0.009).

Risk stratification according to the ESC showed a clear shift toward higher-risk categories as the GmScore increased. Among patients classified as GmS1, 75.6% were categorized as low risk, 24.4% as intermediate–low risk, and none were assigned to the intermediate–high or high-risk groups (0% in both categories). In contrast, patients in the GmS2 and GmS3 categories were predominantly classified as intermediate–high risk (90.0% and 82.9%, respectively). The proportion of high-risk patients increased from 0% in GmS1 to 4.0% in GmS2 and 14.6% in GmS3 (*p* < 0.001; linear trend *p* < 0.001).

### 3.3. Right Ventricular Strain

**Figure 5:**
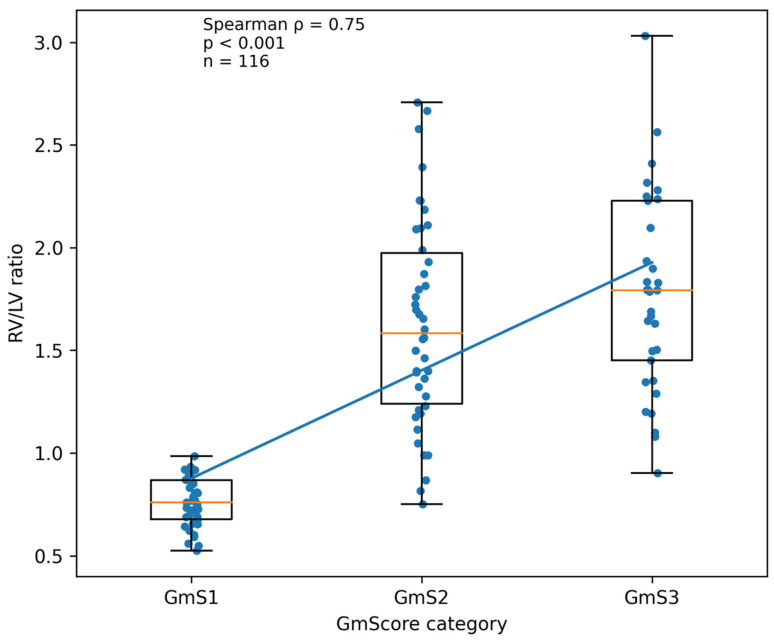
Association between GmScore and the continuous RV/LV ratio. Points represent individual values (with slight horizontal jitter to avoid overlap), and boxplots display the median and interquartile range for each category (Spearman ρ = 0.75; 95% CI: 0.66–0.82; *p* < 0.001; *n* = 116). The solid line illustrates the overall trend. A progressive increase in the RV/LV ratio is observed with higher GmScore categories.

Markers of right ventricular strain followed a consistent progressive pattern (Table 1, Figure 5). The median RV/LV ratio increased from 0.76 (IQR: 0.70–0.82) in GmS1 to 1.58 (IQR: 1.24–1.97) in GmS2 and 1.79 (IQR: 1.45–2.23) in GmS3 (*p* < 0.001, Kruskal–Wallis). Post hoc pairwise comparisons using Dunn’s test with Bonferroni correction demonstrated significant differences between GmS1 and GmS2 (*p* < 0.001) and between GmS1 and GmS3 (*p* < 0.001), whereas no significant difference was observed between GmS2 and GmS3 (*p* = 0.938).

A strong monotonic correlation was observed between GmScore and the continuous RV/LV ratio (Spearman ρ = 0.75; 95% CI: 0.66–0.82; *p* < 0.001; *n* = 116), further supporting the association between anatomical thrombus burden and right ventricular strain.

The proportion of patients with an RV/LV ratio > 1 on CT increased from 0% in GmS1 to 88.0% in GmS2 and 97.6% in GmS3 (*p* < 0.001). Echocardiographic parameters showed a comparable progressive pattern: right ventricular dysfunction was present in 0%, 78.0%, and 92.7%; TAPSE < 16 mm in 0%, 50.0%, and 70.7%; and McConnell’s sign in 0%, 68.0%, and 87.8% of patients in GmS1, GmS2, and GmS3, respectively (all *p* < 0.001).

### 3.4. Dichotomous Analysis: GmS1 Versus GmS2 + GmS3

Post hoc analysis showed that, in terms of right ventricular involvement, GmS2 and GmS3 behaved in a statistically indistinguishable manner (*p* = 0.938), whereas the separation between GmS1 and the higher categories was marked and consistent across all analyzed variables. To further explore the clinical implications of this dichotomization, GmS1 was compared with the combined GmS2 + GmS3 group (Table 3).

No patient classified as GmS1 fell into the intermediate–high or high ESC risk categories, compared with 95.6% in the GmS2 + GmS3 group (95% CI: 89.1–98.8%; *p* < 0.001). Similarly, none of the patients with GmS1 showed an RV/LV ratio > 1 on CT or right ventricular dysfunction on echocardiography, whereas these findings were present in 92.3% and 84.6% of patients in the GmS2 + GmS3 group, respectively (both *p* < 0.001).

Using GmS ≥ 2 as the threshold to identify intermediate–high or high ESC risk, sensitivity was 100.0% (95% CI: 95.8–100.0%), specificity 91.1% (95% CI: 78.8–97.5%), positive predictive value 95.6% (95% CI: 89.1–98.8%), negative predictive value 100.0% (95% CI: 91.4–100.0%), and overall accuracy 97.0%.

### 3.5. Thirty-Day Mortality

Seven deaths from any cause were recorded within the first 30 days (5.3%; 95% CI: 2.2–10.6%). Mortality was 0.0% in GmS1 (0/41; 95% CI: 0.0–8.6%), 2.0% in GmS2 (1/50; 95% CI: 0.1–10.6%), and 14.6% in GmS3 (6/41; 95% CI: 5.6–29.2%).

No deaths occurred among patients with sPESI = 0 or in the low or intermediate–low ESC risk categories. Events were concentrated in the intermediate–high (3/79; 3.8%; 95% CI: 0.8–10.7%) and high-risk strata (4/8; 50.0%; 95% CI: 15.7–84.3%) (Table 4).

## 4. Discussion

In the present study, the GmScore not only showed a progressive variation across anatomical categories but, in practice, behaved as a binary discriminator, with a clear functional separation between GmS1 and the higher categories. This finding has direct implications for its use as a structured descriptor in the radiology report of pulmonary embolism.

Reproducibility indices for the GmScore were high. The interobserver quadratic weighted kappa was 0.92 with a 95% confidence interval of 0.87 to 0.97, and intraobserver agreement was similarly high at 0.92 with a 95% confidence interval of 0.81 to 1.00. These values are consistent with those reported for other CT-based thrombus burden scores [8] and support its applicability in high-pressure clinical settings, where image interpretation may be performed by different readers or at different time points. The fact that all disagreements occurred between adjacent categories, with no discordance between GmS1 and GmS3, further reinforces the internal consistency of the instrument in this cohort.

The differences observed across categories were statistically significant, but the key interpretation lies in the functional asymmetry underlying this gradient. Post hoc analysis showed that GmS2 and GmS3 were indistinguishable in terms of right ventricular involvement, whereas the separation between GmS1 and the higher categories was marked across CT and echocardiographic variables. This pattern reflects the limited capacity of the right ventricle to adapt in a graded manner to acute increases in afterload [3]. Once a critical threshold of obstruction is exceeded, the right ventricle enters a cycle of pressure and volume overload influenced by the speed of onset, pulmonary vasoconstriction, and baseline functional reserve. In this context, the anatomical distinction between lobar and main pulmonary artery involvement may lose functional relevance, potentially explaining the convergence between GmS2 and GmS3. Nevertheless, maintaining three anatomical categories retains clinical value, particularly when considering therapeutic options. Information on the distribution and centrality of thrombus burden may be decisive in emergency scenarios, such as planning aspiration thrombectomy or assessing the indication for surgical embolectomy [12]. This dual nature, three-tiered in anatomical description yet dichotomous in immediate clinical application, is reflected in the performance of GmS ≥ 2 as a negative screening tool for ESC intermediate–high or high risk in this cohort, with a sensitivity of 100.0% and a negative predictive value of 100.0%.

The 30-day mortality rate in this cohort was 5.3%, which falls within the range reported in international pulmonary embolism registries [13]. No deaths occurred in patients classified as GmS1, whereas mortality reached 14.6% in GmS3, and 50.0% in the high ESC risk stratum. Although the number of events was insufficient to draw independent prognostic conclusions, this distribution is consistent with the anatomical and hemodynamic severity observed in the higher GmScore categories and may support the construct validity of the descriptor.

The choice of the Ghanima score as a starting point was guided by considerations of clinical practicality. Segment-based models such as Mastora or Qanadli allow detailed quantification of pulmonary arterial obstruction but require systematic assessment that may be less feasible in emergency settings [9,14,15,16]. In a comparative analysis of 50 patients, Meyer and colleagues demonstrated significant differences in calculation time: the Mastora index required approximately 300 s per case, compared with approximately 90 s for the Qanadli index and 25 s for the Ghanima score, with all pairwise comparisons reaching statistical significance (*p* < 0.0001) [8]. Interobserver agreement was excellent and statistically indistinguishable across all scores [8]. Of note, the Ghanima score showed only moderate correlation with the segment-based models, suggesting it captures a partially distinct dimension of thrombus burden [8]. However, proximal location alone does not reliably reflect hemodynamic impact. In the TROLL study, no significant differences in mortality were observed between central and peripheral emboli [7], suggesting that proximal extension by itself does not fully capture the clinical relevance of the episode. By incorporating a threshold of luminal occupation of at least 50%, the modified GmScore directly addresses this limitation, requiring not only the presence of thrombus at a given anatomical level but also sufficient obstruction to translate into increased right ventricular afterload [3]. The result is a descriptor that combines the operational efficiency of the original model with a semiquantitative component grounded in pathophysiology, which may make it particularly suitable for integration into emergency radiology reporting.

From a clinical standpoint, the GmScore is not intended to replace established risk stratification tools, but to complement them. Scores such as PESI and sPESI have demonstrated adequate performance in identifying low-risk patients suitable for outpatient management [17,18]. In our cohort, the alignment between GmScore and ESC risk stratification was clear and bidirectional. Most patients classified as low or intermediate–low risk corresponded to GmS1, whereas all intermediate–high and high-risk patients were classified as GmS2 or GmS3. This alignment suggests its potential utility as a structured descriptor that facilitates and enriches communication between radiologists and clinicians during the initial evaluation.

The categorical and hierarchical nature of the GmScore also makes it particularly compatible with automated workflows. Artificial intelligence algorithms are already capable of detecting pulmonary embolism and estimating the RV-to-LV ratio on CT pulmonary angiography [19], and recent advances in foundation model-based approaches have demonstrated the feasibility of automated three-dimensional thrombus segmentation and burden quantification directly from CTPA [20]. In this context, the distinction between GmS1 and GmS2 or higher would substantially simplify automated classification. Compared with segment-based models, which require individualized quantification of multiple arterial branches, the GmScore reduces the task to identifying the most proximal vessel with clinically relevant obstruction and assigning a binary output. This structure is compatible with rule-based or supervised learning decision architectures and could be integrated into automated reporting systems, promoting standardization in pulmonary embolism communication without adding complexity to the workflow.

This study has several limitations. Its retrospective and single-center design may limit generalizability to other healthcare settings. The study was not designed to develop an independent predictive model, and the number of mortality events was insufficient for multivariable analysis. External validation in independent cohorts will be necessary to confirm the reproducibility and applicability of the GmScore in different clinical contexts. Additionally, the number of studies screened and excluded during the recruitment period was not prospectively recorded, which prevents reporting a complete participant flow. Finally, echocardiographic data were not obtained at a uniform time point, as patients at lower risk were assessed within the first 72 h. This timing may introduce bias, as parameters obtained earlier could reflect a more acute haemodynamic state.

In conclusion, the present data suggest that the modified GmScore may serve as a simple, reproducible, and pathophysiologically coherent radiological descriptor of thrombus burden in acute pulmonary embolism. Its application appears to allow an operational distinction between low thrombus burden represented by GmS1 and high thrombus burden represented by GmS2 or higher, with consistent alignment with right ventricular involvement and clinical risk stratification in this cohort. These findings support its potential role as a structured anatomical descriptor to enhance communication between radiology and clinical teams during initial patient assessment. Integration into automated reporting workflows within pulmonary embolism response systems represents a potential direction for future research, requiring prospective evaluation.

## Figures and Tables

**Table 1:** Protocol for planning a CTPA for the study of pulmonary thromboembolism.

	General Electric (Revolution EVO)
**Scan mode**	Single energy (128)
**Scan area**	Diaphragm to lung apex
**Scan direction**	Caudo-cranial
**Scan time (s)**	3.32 s
**Tube voltage (kVp)**	100
**Tube current (ref. mAs)**	130
**CTDIvol (mGy)**	8.5
**Rotation time (s)**	0.4
**Pitch**	0.98
**Slice collimation (mm)**	0.625
**Acquisition (mm)**	128 × 0.4
**Iodine concentration**	300 mg/mL
**Contrast media volume (mL/kg)**	1.5
**Contrast media flow rate (ml/s)**	4
**Bolus timing**	Bolus tracking
**Bolus tracking threshold (HU)**	100
**ROI position**	Pulmonary trunk
**Scan delay (s)**	6
**Saline flush volume (mL)**	40
**Saline injection rate (mL/s)**	4
**Needle size (G)**	18
**Injection site**	Antecubital vein

CTDI (computed tomography dose index); s (seconds); kVp (kilovoltage peak); mAs (milliampere-seconds); mGy (miliGray); HU (Hounsfield Units); ROI (Region of Interest); G (Gauge). Revolution EVO (GE HealthCare, Chicago, IL, USA).

**Table 2:** Clinical and imaging characteristics according to GmScore.

Variable	GmS1 (N = 41)	GmS2 (N = 50)	GmS3 (N = 41)	*p*-Value
**Age (years)**	65.7 ± 18.7	64.9 ± 15.0	63.8 ± 16.4	0.806
**Male sex**	27 (65.9%)	27 (54.0%)	23 (56.1%)	0.491
**sPESI ≥ 1**	29 (70.7%)	43 (86.0%)	39 (95.1%)	0.009
**ESC** **risk category**				<0.001
Low	31 (75.6%)	0 (0.0%)	0 (0.0%)	
Intermediate–low	10 (24.4%)	3 (6.0%)	1 (2.4%)	
Intermediate–high	0 (0.0%)	45 (90.0%)	34 (82.9%)	
High	0 (0.0%)	2 (4.0%)	6 (14.6%)	
**RV/LV** ratio (CT) *	0.76 ± 0.12	1.63 ± 0.51	1.78 ± 0.48	<0.001
**RV/LV > 1 (CT)**	0 (0.0%)	44 (88.0%)	40 (97.6%)	<0.001
**Right ventricular dysfunction (echo)**	0 (0.0%)	39 (78.0%)	38 (92.7%)	<0.001
**TAPSE < 16** **mm**	0 (0.0%)	25 (50.0%)	29 (70.7%)	<0.001
**M** **cConnell’s sign**	0 (0.0%)	34 (68.0%)	36 (87.8%)	<0.001
**30-** **day mortality**	0 (0.0%)	1 (2.0%)	6 (14.6%)	0.005

Continuous variables are presented as mean ± standard deviation (SD) or median (interquartile range), as appropriate. Categorical variables are expressed as number of patients (percentage within each GmScore category). *p*-values are reported for variables tested for association with GmScore categories. Continuous variables were compared using the Kruskal–Wallis test. Dichotomous categorical variables were compared using Fisher’s exact test. The ESC risk category (4-category ordinal variable) was compared using the Freeman–Halton exact test. * The continuous RV/LV ratio was available in 116 patients. sPESI: simplified Pulmonary Embolism Severity Index; ESC: European Society of Cardiology; RV/LV: right-to-left ventricular diameter ratio; CT: computed tomography; TAPSE: tricuspid annular plane systolic excursion; SD: standard deviation.

**Table 3:** Comparison of clinical and imaging variables between GmS1 and GmS2 + GmS3.

Variable	GmS1 (N = 41)	GmS2 + GmS3 (N = 91)	*p*-Value
**ESC** **intermediate–high/high**	0/41 (0.0%; 95%CI: 0.0–8.6%)	87/91 (95.6%; 95%CI: 89.1–98.8%)	<0.001
**sPESI ≥ 1**	29/41 (70.7%; 95%CI: 54.5–83.9%)	82/91 (90.1%; 95%CI: 82.1–95.4%)	0.009
**RV/LV > 1 (CT)**	0/41 (0.0%; 95%CI: 0.0–8.6%)	84/91 (92.3%; 95%CI: 84.8–96.9%)	<0.001
**RV** **dysfunction (ECHO)**	0/41 (0.0%; 95%CI: 0.0–8.6%)	77/91 (84.6%; 95%CI: 75.5–91.3%)	<0.001
**TAPSE < 16** **mm**	0/41 (0.0%; 95%CI: 0.0–8.6%)	54/91 (59.3%; 95%CI: 48.5–69.5%)	<0.001
**M** **cConnell’s sign**	0/41 (0.0%; 95%CI: 0.0–8.6%)	70/91 (76.9%; 95%CI: 66.9–85.1%)	<0.001
**Mortality < 30 days**	0/41 (0.0%; 95%CI: 0.0–8.6%)	7/91 (7.7%; 95%CI: 3.1–15.2%)	0.098

Categorical variables are expressed as the number of patients (percentage; 95% confidence intervals calculated using the exact Clopper–Pearson method). *p* values correspond to Fisher’s exact test. RV: right ventricle; ESC: European Society of Cardiology; RV/LV: right-to-left ventricular ratio; CT: computed tomography; TAPSE: tricuspid annular plane systolic excursion; sPESI: simplified Pulmonary Embolism Severity Index.

**Table 4:** Thirty-day mortality according to GmScore, sPESI, and ESC classification.

Variable	Category	Deaths *n*/N (%)	95% CI
**Overall**	—	**7/132 (5.3%)**	**2.2–10.6%**
**G** **mSCORE**	GmS1	0/41 (0.0%)	0.0–8.6%
	GmS2	1/50 (2.0%)	0.1–10.6%
	GmS3	6/41 (14.6%)	5.6–29.2%
**sPESI**	0	0/21 (0.0%)	0.0–16.1%
	≥1	7/111 (6.3%)	2.6–12.6%
**ESC** **risk**	Low	0/31 (0.0%)	0.0–11.2%
	Intermediate–low	0/14 (0.0%)	0.0–23.2%
	Intermediate–high	3/79 (3.8%)	0.8–10.7%
	High	4/8 (50.0%)	15.7–84.3%

Data are presented as number of deaths over total number of patients within each category (percentage). The 95% confidence intervals were calculated using the Clopper–Pearson exact method. sPESI: simplified Pulmonary Embolism Severity Index; ESC: European Society of Cardiology.

## Data Availability

The data supporting the findings of this study are available from the corresponding author upon reasonable request.

## Supplementary Materials

The following supporting information can be downloaded at: https://www.mdpi.com/article/10.3390/jimaging12070327/s1, Table S1: STROBE Statement checklist for cohort studies.
