# Supplementary material for: A Simplified CT Score for Thrombus Burden in Acute Pulmonary Embolism: Clinical Correlation and Reproducibility
Source: J Imaging. 2026 Jul 19;12(7):327. doi: 10.3390/jimaging12070327 (PMC13412854; doi:10.3390/jimaging12070327)
Supplement: Supplementary file 1 [file jimaging-12-00327-s001.zip › jimaging-4365821-supplementary.pdf]

Table S1. STROBE Statement—checklist of items that should be included in reports of observational studies

Manuscript: “A Simplified CT Score for Thrombus Burden in Acute Pulmonary Embolism: Clinical Correlation and Reproducibility”

	Item No.	Recommendation	Page No.	Relevant text from manuscript
**Title and abstract**				
**Title and abstract**	1	(a) Indicate the study's design with a commonly used term in the title or the abstract	1	The title conveys the study type through its descriptive framing: "A Simplified CT Score for Thrombus Burden in Acute Pulmonary Embolism: Clinical Correlation and Reproducibility." The abstract reinforces this in the opening sentence: "In this retrospective single centre study."
		(b) Provide in the abstract an informative and balanced summary of what was done and what was found	1	The abstract is structured across four clearly labelled sections: Background, Methods, Results and Conclusion. Key numerical findings are reported throughout: interobserver kappa 0.92, Spearman rho 0.75, 100% sensitivity and 100% negative predictive value for GmS>=2 in detecting intermediate to high and high ESC risk. The conclusion frames the score as a practical descriptor rather than a standalone prognostic tool, which reflects the study's actual scope.
**Introduction**				
**Background/ rationale**	2	Explain the scientific background and rationale for the investigation being reported	2	The rationale is built on three converging arguments: narrative radiology reports introduce variability that limits clinical utility in time pressured settings; PE management depends on risk stratification that integrates imaging data, particularly the RV/LV ratio; and the existing clot burden scores are too complex for routine urgent use. The gap these observations define is precisely what the GmScore is designed to address.
**Objectives**	3	State specific objectives, including any prespecified hypotheses	2, 3	The aim is to evaluate a modified version of the Ghanima score, which was predefined at study conception as a registry variable, across two specific endpoints: its clinical correlation with established risk stratification instruments (ESC category, sPESI, RV/LV ratio and echocardiographic RV dysfunction) and its reproducibility expressed as inter and intraobserver agreement.
**Methods**				
**Study design**	4	Present key elements of study design early in the paper	3, 4	Stated at the outset of the Methods section: "This is a retrospective, single-center observational study based on a systematic patient registry, in which study variables, including the GmScore definition, were established at the time of study conception." The prospective registry framework is emphasised to distinguish the work from an unstructured retrospective chart review.
**Setting**	5	Describe the setting, locations, and relevant dates, including periods of recruitment, exposure, follow-up, and data collection	4	Single academic centre. CT pulmonary angiography studies performed between January 2021 and November 2025 were reviewed according to a prespecified structured protocol. Ethics committee approval was obtained and informed consent was waived given the retrospective nature of the study. Patients were enrolled consecutively, with recruitment in each GmScore category closed once the predefined sample size was reached.
**Participants**	6	(a) Cohort study: give the eligibility criteria, and the sources and methods of selection of participants. Describe methods of follow-up	3, 4	Adults with confirmed acute PE on CT pulmonary angiography were enrolled consecutively. Studies were excluded when technical quality was insufficient for GmScore classification, and cases of chronic PE or incidental PE were also excluded. All patients were followed for 30 days for the mortality outcome. ESC risk stratification and sPESI were assigned by the treating physician at initial assessment. Echocardiographic data were obtained within the first 72 hours in low and intermediate low risk patients, and immediately in those with intermediate to high or high risk.
		(b) Cohort study: for matched studies, give matching criteria and number of exposed and unexposed	N/A	No matching was applied. This is an unmatched cohort stratified by anatomical imaging category.

**Variables**	7	Clearly define all outcomes, exposures, predictors, potential confounders, and effect modifiers. Give diagnostic criteria, if applicable	3, 5	The exposure variable, GmScore, is defined in full: three ordinal categories (GmS1: segmental, GmS2: lobar, GmS3: main pulmonary arteries) based exclusively on filling defects with 50% or greater luminal obstruction, prioritising the most proximal affected vessel regardless of the number of branches involved. Outcomes comprise ESC risk category (four level ordinal variable), sPESI of 1 or above (binary), CT RV/LV ratio above 1 (binary and continuous), echocardiographic RV dysfunction (TAPSE below 16 mm, McConnell sign), and 30 day all cause mortality. All diagnostic thresholds are referenced to published guidelines and prior validation studies.
**Data sources/ measurement**	8	For each variable of interest, give sources of data and details of methods of assessment (measurement). Describe comparability of assessment methods if there is more than one group	3, 4	A structured report template was applied consistently across all cases, covering: description of filling defects and degree of luminal obstruction; GmScore category; and axial RV/LV ratio. The RV/LV ratio was measured as the maximum diameter of each ventricle on the axial slice showing the largest cross section. Echocardiographic parameters were retrieved from clinical cardiology reports. Identical imaging protocols and measurement criteria were used across all three GmScore groups.
**Bias**	9	Describe any efforts to address potential sources of bias	3, 4	At the time of the first reading, patients were still in the diagnostic workup phase: ESC risk stratification had not yet been established, so readers were effectively unaware of the clinical outcome when assigning GmScore categories. For the intraobserver assessment, although risk stratification was by then available, it was not consulted during image review. Biochemical and echocardiographic data were not accessible to either reader at any point during GmScore classification. Interobserver agreement was assessed across the full dataset; intraobserver reproducibility was evaluated in a randomly selected subset of 30 cases with a minimum washout interval of four weeks.
**Study size**	10	Explain how the study size was arrived at	5	No formal power calculation was conducted for hypothesis testing. The target of 30 to 40 patients per group was instead based on the requirements for stable association estimates [ref. 11] and a reliable weighted kappa coefficient with three ordinal categories [ref. 12]. The final cohort comprised 41 patients in GmS1, 50 in GmS2 and 41 in GmS3, totalling 132.
**Quantitative variables**	11	Explain how quantitative variables were handled in the analyses. If applicable, describe which groupings were chosen and why	3, 5	All thresholds were prespecified in the study protocol and grounded in prior evidence: 50% luminal obstruction (Mastora et al., associated with significantly higher mean pulmonary arterial pressure); RV/LV ratio above 1 (ESC guidelines for RV dilation); sPESI of 1 or above (standard original definition); TAPSE below 16 mm (echocardiographic criterion for RV dysfunction). The GmScore was analysed as a three category ordinal variable in primary analyses and as a binary variable (GmS1 versus GmS>=2) in the secondary analysis.
**Statistical methods**	12	(a) Describe all statistical methods, including those used to control for confounding	5	Continuous variables across GmScore categories were compared using the Kruskal Wallis test, with pairwise post hoc comparisons by Dunn's test with Bonferroni correction. Dichotomous categorical variables were analysed with Fisher's exact test. The four category ESC risk variable was tested using the Freeman Halton exact test. Linear trend across ordered categories was assessed with the Cochran Armitage test. Association between GmScore and the continuous RV/LV ratio was quantified by Spearman rank correlation. Diagnostic performance metrics were computed with 95% confidence intervals by the Clopper Pearson exact method. Observer agreement was assessed by quadratic weighted kappa with 95% confidence interval. All analyses were performed in Python 3.12.12 (Python Software Foundation). Statistical significance was set at p below 0.05.

		(b) Describe any methods used to examine subgroups and interactions	5, 8	A secondary analysis dichotomised the GmScore into GmS1 versus GmS2 plus GmS3, based on the post hoc finding that GmS2 and GmS3 were statistically indistinguishable for ventricular repercussion (p = 0.938). Sensitivity, specificity, positive predictive value and negative predictive value with 95% confidence intervals were calculated for this binary threshold in identifying intermediate to high and high ESC risk. Mortality was also tabulated separately by sPESI and ESC risk category (Table 3).
		(c) Explain how missing data were addressed	5, 6	Missing data were confined to the continuous RV/LV ratio, unavailable in 16 of 132 patients (12%), all of whom had been referred from an external centre where images could be reviewed but not measured. These cases are described explicitly. Analyses of the continuous RV/LV ratio report n = 116. No imputation was performed. All remaining study variables were complete across the full cohort.
		(d) Cohort study: if applicable, explain how loss to follow-up was addressed	N/A	There was no loss to follow up. All 132 included patients completed the 30 day observation window for the mortality endpoint.
		(e) Describe any sensitivity analyses	8, 9	The binary analysis (GmS1 versus GmS>=2) serves as the primary applicability check: it evaluates whether collapsing the three level score into a single threshold preserves, or even sharpens, clinical discriminative performance. Sensitivity: 100.0% (95% CI: 95.8 to 100.0%); negative predictive value: 100.0% (95% CI: 91.4 to 100.0%); specificity: 91.1% (95% CI: 78.8 to 97.5%); positive predictive value: 95.6% (95% CI: 89.1 to 98.8%); overall accuracy: 97.0%.
**Results**				
**Participants**	13	(a) Report numbers of individuals at each stage of study: numbers potentially eligible, examined for eligibility, confirmed eligible, included in the study, completing follow-up, and analysed	5, 6	144 studies reviewed; 12 excluded due to insufficient technical quality for GmScore classification, A total of 132 consecutive patients with confirmed acute PE were included and analysed: GmS1 n = 41, GmS2 n = 50, GmS3 n = 41. The number of studies reviewed and rejected during the recruitment period was not prospectively recorded. All 132 patients completed follow-up.
		(b) Give reasons for non-participation at each stage	5, 6	The exclusion criterion applied was insufficient technical quality for GmScore classification. Twelve studies were excluded on this basis. The absence of a total screened number is acknowledged as a methodological limitation of the quota-based design and is addressed in the Limitations section.
		(c) Consider use of a flow diagram	5,6	A flow diagram is provided as Figure 2, including the number of cases reviewed (n = 144), excluded (n = 12), and included in the final cohort (n = 132)..
**Descriptive data**	14	(a) Give characteristics of study participants (e.g. demographic, clinical, social) and information on exposures and potential confounders	6, Table 1	Table 1 reports participant characteristics by GmScore category: age (mean and standard deviation), sex, sPESI of 1 or above, ESC risk distribution, mean and binary RV/LV ratio on CT, echocardiographic RV dysfunction, TAPSE below 16 mm, McConnell sign and 30 day mortality. Baseline demographics were balanced: no significant differences in mean age (65.7 vs. 64.9 vs. 63.8 years; p = 0.806) or sex distribution (p = 0.491) across groups.
		(b) Indicate number of participants with missing data for each variable of interest	5, 6	Reported in the Results section and as a footnote to Table 1: the continuous RV/LV ratio was available in 116 of 132 patients (88%). All other study variables were complete for the full cohort.
		(c) Cohort study: summarise follow-up time (e.g. average and total amount)	N/A	All patients were observed for a fixed period of 30 days, with no variation in duration and no losses. Summarising average observation time is therefore not applicable.
**Outcome data**	15	Cohort study: report numbers of outcome events or summary measures over time	6, Tables 1 to 3	Thirty day all cause mortality: 7 out of 132 patients (5.3%; 95% CI: 2.2 to 10.6%). By GmScore: GmS1, 0 out of 41 (0.0%); GmS2, 1 out of 50 (2.0%; 95% CI: 0.1 to 10.6%); GmS3, 6 out of 41 (14.6%; 95% CI: 5.6 to 29.2%). No deaths occurred in patients with sPESI = 0 or in low or intermediate low

				ESC risk groups. Mortality reached 50.0% (95% CI: 15.7 to 84.3%) in the high risk ESC stratum. Table 1 provides event rates for all binary imaging and clinical outcomes by GmScore category.
**Main results**	16	(a) Give unadjusted estimates and, if applicable, confounder-adjusted estimates and their precision (e.g. 95% confidence interval). Make clear which confounders were adjusted for and why they were included	7 to 10	All results are unadjusted, which is consistent with the descriptive and associative design: no multivariable predictive model was developed or intended. Estimates are accompanied by 95% confidence intervals throughout. Spearman rho = 0.75 (95% CI: 0.66 to 0.82; p below 0.001; n = 116). Interobserver weighted kappa = 0.92 (95% CI: 0.87 to 0.97); intraobserver kappa = 0.92 (95% CI: 0.81 to 1.00). Full diagnostic performance metrics for GmS>=2 are reported under item 12e.
		(b) Report category boundaries when continuous variables were categorized	3, 5	All thresholds are stated explicitly in the Methods and supported by cited prior work: luminal obstruction of 50% or more (Mastora et al.), RV/LV ratio above 1 (ESC guidelines), sPESI of 1 or above (original derivation study), TAPSE below 16 mm (standard echocardiographic criterion for RV dysfunction).
		(c) If relevant, consider translating estimates of relative risk into absolute risk for a meaningful time period	N/A	Relative risk estimates were not computed. The study reports association measures (Spearman rho, weighted kappa) and diagnostic performance metrics (sensitivity, specificity, positive and negative predictive values). Translation to absolute risk is therefore not applicable.
**Other analyses**	17	Report other analyses done: analyses of subgroups and interactions, and sensitivity analyses	7 to 10	Post hoc Dunn pairwise comparisons with Bonferroni correction for the continuous RV/LV ratio: GmS1 versus GmS2 (p below 0.001); GmS1 versus GmS3 (p below 0.001); GmS2 versus GmS3 (p = 0.938). Secondary binary analysis comparing GmS1 against GmS2 plus GmS3, with full diagnostic performance metrics (Table 2). Mortality tabulated separately by GmScore category, sPESI and ESC risk class (Table 3).
**Discussion**				
**Key results**	18	Summarise key results with reference to study objectives	10	The Discussion opens by identifying the central finding: rather than a simple ordinal gradient, the GmScore behaves functionally as a binary discriminator, with a clear separation between GmS1 and the two higher categories. GmS2 and GmS3 are statistically indistinguishable for RV repercussion, a pattern the authors attribute to the limited capacity of the right ventricle to adapt in a graded manner to acute pressure overload.
**Limitations**	19	Discuss limitations of the study, taking into account sources of potential bias or imprecision. Discuss both direction and magnitude of any potential bias	12	Five main limitations are acknowledged: (1) retrospective single-center design, which may limit generalisability to other settings; (2) the study was not sized or designed for independent prognostic modelling, and the number of deaths was too small for multivariable analysis; (3) external validation in independent cohorts will be necessary; (4) the number of studies screened and excluded during the recruitment period was not prospectively recorded, partially addressed in the revised manuscript by reporting the total number of reviewed cases (n = 144) and excluded studies (n = 12); and (5) the potential bias introduced by the non-uniform timing of echocardiographic assessment across risk groups. All limitations are explicitly stated in the Limitations paragraph.
**Interpretation**	20	Give a cautious overall interpretation of results considering objectives, limitations, multiplicity of analyses, results from similar studies, and other relevant evidence	10 to 12	The GmScore is framed as a structured descriptor intended to complement, not replace, established clinical scales. Its alignment with ESC risk stratification is characterised as bidirectional and consistent, while the convergence of GmS2 and GmS3 is interpreted through RV physiology rather than treated as a shortcoming of the score. The authors are explicit that independent prognostic validation requires prospective studies with larger event numbers.
**Generali-sability**	21	Discuss the generalisability (external validity) of the study results	12	External validation in independent cohorts is explicitly requested: "A prospective external validation in independent cohorts will be necessary to confirm the reproducibility and applicability of the GmScore in other clinical

				settings." The single centre design is recognised as a factor that may limit how broadly the results can be transferred.
**Other information**				
**Funding**	22	Give the source of funding and the role of the funders for the present study and, if applicable, for the original study on which the present article is based	Title page	Declared in the editorial submission form (title page) in accordance with Insights into Imaging requirements. Not included in the main manuscript body. This research received no external funding

*Give information separately for cases and controls in case-control studies and, if applicable, for exposed and unexposed groups in cohort and cross-sectional studies.
